# The setting of the rising sun? A recent comparative history of life expectancy trends in Japan and Australia

**DOI:** 10.1371/journal.pone.0214578

**Published:** 2019-03-28

**Authors:** Tim Adair, Rebecca Kippen, Mohsen Naghavi, Alan D. Lopez

**Affiliations:** 1 Melbourne School of Population and Global Health, The University of Melbourne, Carlton, Victoria, Australia; 2 School of Rural Health, Monash University, Victoria, Australia; 3 Institute for Health Metrics and Evaluation, Seattle, Washington, United States; University of Taipei, TAIWAN

## Abstract

**Introduction:**

Adult male and female mortality declines in Japan have been slower than in most high-income countries since the early 1990s. This study compares Japan’s recent life expectancy trends with the more favourable trends in Australia, measures the contribution of age groups and causes of death to differences in these trends, and places the findings in the context of the countries’ risk factor transitions.

**Methods:**

The study utilises data on deaths by age, sex and cause in Australia and Japan from 1950–2016 from the Global Burden of Disease Study. A decomposition method measures the contributions of various ages and causes to the male and female life expectancy gap and changes over four distinct phases during this period. Mortality differences by cohort are also assessed.

**Findings:**

Japan’s two-year male life expectancy advantage over Australia in the 1980s closed in the following 20 years. The trend was driven by ages 45–64 and then 65–79 years, and the cohort born in the late 1940s. Over half of Australia’s gains were from declines in ischaemic heart disease (IHD) mortality, with lung cancer, chronic respiratory disease and self-harm also contributing substantially. Since 2011 the trend has reversed again, and in 2016 Japan had a slightly higher male life expectancy. The advantage in Japanese female life expectancy widened over the period to 2.3 years in 2016. The 2016 gap was mostly from differential mortality at ages 65 years and over from IHD, chronic respiratory disease and cancers.

**Conclusions:**

The considerable gains in Australian male life expectancy from declining non-communicable disease mortality are attributable to a range of risk factors, including declining smoking prevalence due to strong public health interventions. A recent reversal in life expectancy trends could continue because Japan has greater scope for further falls in smoking and far lower levels of obesity. Japan’s substantial female life expectancy advantage however could diminish in future because it is primarily due to lower mortality at old ages.

## Introduction

Japan’s economic expansion in the post-World War II era was characterised by phenomenal increases in survivorship. Over the period 1950–85, Japanese life expectancy at birth increased by 20 years for females and 18 years for males, leapfrogging life expectancies in other developed countries, such as Australia. By the late 1980s, both male and female Japanese life expectancy was the highest in the world [1].

However, since the early 1990s Japanese mortality declines among males and, to a lesser extent, among adult females, have been slower than observed for most other high-income countries [2]. While much research has detailed the reasons behind the strong increases in Japanese life expectancy in the decades following World War Two, less is known about why it is has slowed more recently. Recent research has identified several health challenges for Japan, including relatively high smoking rates, an increase in body-mass index, and relatively high suicide rates; the latter being linked with declining employment rates due to economic stagnation [3–5].

Despite these unfavourable recent trends, Japan is widely reported as having the highest life expectancy in the world, which for males is no longer the case [6]. It is therefore of interest to closely compare Japanese life expectancy trends with other high-income countries in order to assess how unique the post-war Japanese epidemiological trajectory has been, and where it might be headed. We have chosen Australia as a comparator nation, where mortality trends in recent decades have been particularly favourable, not only compared with Japanese males, but also compared with most other high-income countries [2]. A number of studies have examined the strong declines in Australian mortality rates since 1970, especially for males, attributing these increases to declines in mortality from bold public health interventions which have dramatically reduced death rates from leading causes such as ischaemic heart disease, stroke, other smoking-related causes and road traffic accidents [7–10].

In this paper, we report on a comprehensive comparative study of recent life expectancy trends between Japan and Australia, providing a population health perspective on the impact of disease and risk factor control programs in both countries. We identify the contribution of age groups and causes of death to differences in Japanese and Australian life expectancy trends since 1950, taking a birth cohort perspective. We identify four periods over the past seven decades or so that approximate distinct phases of the comparative relationship in Japanese and Australian life expectancy, and examine the importance of differential mortality changes at various ages and from various causes of death to observed changes in life expectancy within these periods. We place these trends in the context of risk factor transitions in both countries and offer some insights into the future of mortality decline in both countries.

## Methods

### Data

We obtained data on deaths by five-year age group, sex and cause, and population by five-year age group and sex, in Australia and Japan from 1950 to 2016 from the Global Burden of Disease (GBD) Study [2]. The GBD Study makes a number of adjustments to vital registration data to address quality issues introduced by certification practices and other issues between countries and across time. For example, according to vital registration data, dementia death rates per 100,000 population at 85 years and above are six times higher in Australia than in Japan, which is clearly a result of different certification practices.[11] Data for the period 1980–2016 underwent the GBD adjustment process, which includes: re-assigning ‘garbage’ or impossible causes of death to other causes (such as senility, heart failure or septicaemia) to improve the specificity and diagnostic accuracy of the cause of death data; ‘noise reduction’ to handle problems of zero deaths for specific age-sex-cause combinations; estimating mortality for some causes with notable data quality issues, such as dementia, using prevalence and other data sources; and application of the CoDCorrect algorithm to proportionately adjust cause-specific estimates to fit all-cause estimates, by age and sex, based on the relative uncertainty around the cause-specific estimates.[12, 13] Data from 1950–79 also underwent the ‘garbage re-assignment’ process; however noise reduction and the CoDCorrect algorithm could not be applied to these data because they were based on earlier versions of the International Classification of Diseases. These adjustments result in re-estimation of death numbers from vital registration for every cause of death. The GBD cause list, based on extensive public health consultation and scrutiny, and including over 300 diseases and injuries, was used for this analysis; we grouped these into broader causes for our analysis, for which the ICD codes are shown in S1 Text [14]. We excluded deaths from ‘Exposure to forces of nature, disaster’ in our analysis, to remove the effect of natural disasters in Japan, particularly the significant impact of the 2011 earthquake and tsunami.

### Methods

A convenient summary technique to assess and compare the contributions of mortality differences at different ages and from different causes to the gap in life expectancy between two populations is the life table decomposition method developed by Arriaga and Pollard [15,16]. Using this method, the contribution of mortality differences within age groups to the life expectancy gap was calculated as:

Δnx=lx1l01.(Lnx2lx2-Lnx1lx1)+Tx+n2l01.(lx1lx2-lx+n1lx+n2)

Δ∞x=lx1l01.(Tx2lx2-Tx1lx1)

e00(2)-e00(1)=∑xΔnx

where _*n*_Δ_*x*_ is the contribution of mortality difference in age group *x* to *x* + *n* to differences in life expectancy at birth, _∞_Δ_*x*_ is the contribution of mortality difference in the open-ended age group to differences in life expectancy at birth, $l_{x}^{1}$, Lnx1 and $T_{x}^{1}$ are life table functions in the Japanese population, $l_{x}^{2}$, Lnx2 and $T_{x}^{2}$ are life table functions in the Australian population, $e_{0}^{0}(2)$ is life expectancy at birth in the Australian population and $e_{0}^{0}(1)$ is life expectancy at birth in the Japanese population.[17] Importantly, the sum of the contribution of age groups to the life expectancy gap between Australia and Japan will equal the total life expectancy gap.

**Fig 1:**
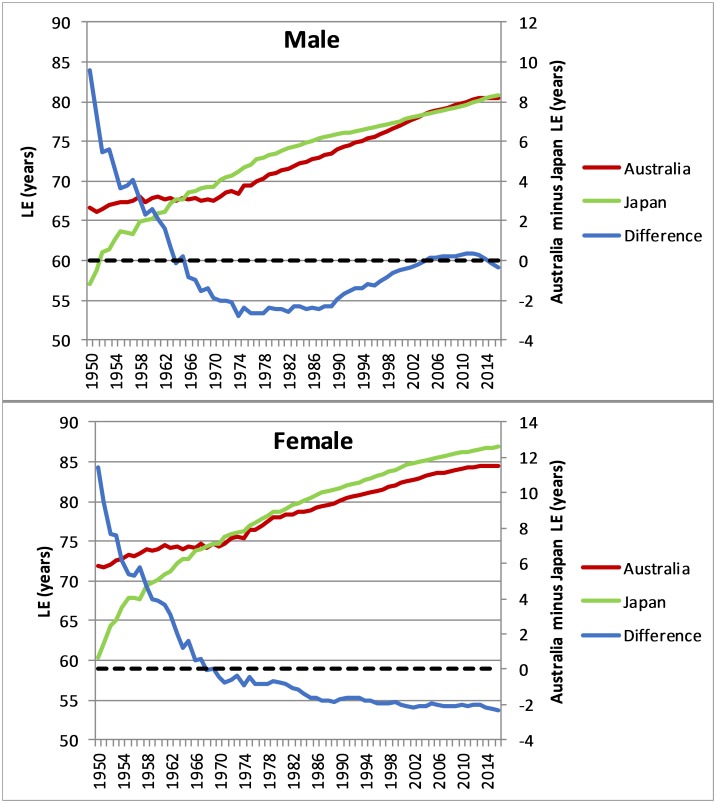
Life expectancy at birth, males and females, Australia, Japan and difference, 1950–2016.

Decomposition of the contribution of causes of death to life expectancy was computed as follows:

Δnxi=Δnx.(Rnxi(2).mnx(2)-Rnxi(1).mnx(1)mnx(2)-mnx(1))

where Δnxi is the contribution of cause *i* in age groups *x* to *x* + *n* to differences in life expectancy at birth, _*n*_Δ_*x*_ is the contribution of all-cause mortality difference in age groups *x* to *x* + *n* to differences in life expectancy at birth (as above), _*n*_*m*_*x*_(1) and _*n*_*m*_*x*_(2) are the all-cause mortality rates in age groups *x* to *x* + *n* in the Japanese and Australian population respectively and Rnxi(1) and Rnxi(2) are the proportion of deaths from cause *i* in age groups *x* to *x* + *n* in the Japanese and Australian population respectively [17]. We applied these methods to Japanese and Australian mortality data at five points in time: 1951, 1970, 1990, 2011 and 2015 (using 3-year averages around each date to smooth variability in life expectancy differences), in order to measure trends in the age-cause contributions to the changing gap in life expectancy between the two countries. These periods were chosen because they identify distinct phases in the evolution of the life expectancy gap between Australia and Japan (see Fig 1). The age and cause contributions to the observed change in the life expectancy gap over a period was calculated by subtracting the contributions at the period start point from the respective contributions at the period end point. The age and cause contribution to the change in the life expectancy gap over a period was measured as the contribution at the end of a period minus the respective contribution at the start of a period. This approach attributes change in relative life expectancy during each period to change in the relative contribution of each age group or each cause. Age groups or causes that contribute to increases in Australia’s life expectancy compared with Japan would have favourable mortality trends in those age groups or causes in Australia versus Japan. We also calculated the ratio of Australian to Japanese all-cause age-specific death rates by cohort year of birth, given the dominant role of these conditions in mortality levels and trends in both countries. The cohort year of birth is the cohort mid-point, calculated as the five-year period minus the five-year age-specific death rate (for example, age group 75–79 in period 2012–16 has a cohort year of birth of 1937).

## Results

### Life expectancy

Australia’s male life expectancy advantage of almost 10 years in 1950 reversed dramatically in the following two-and-a-half decades, as Japanese male life expectancy rose sharply and Australia’s stagnated (Fig 1). However, from the mid-1970s, Australia’s male life expectancy began increasing at the same rate as in Japan, and from the early 1990s, began closing the gap with Japan, where male life expectancy increases had slowed. By 2011 male life expectancy in Australia slightly exceeded that in Japan, but since then the trend has reversed with Japan having a marginally higher life expectancy than Australia in 2016. Relative trends in female life expectancy from 1950–75 were similar as for males, however in this case the Japanese advantage continued to widen to 2016 (2.3 years), the widest gap of the period.

### Contribution of age groups

**Fig 2:**
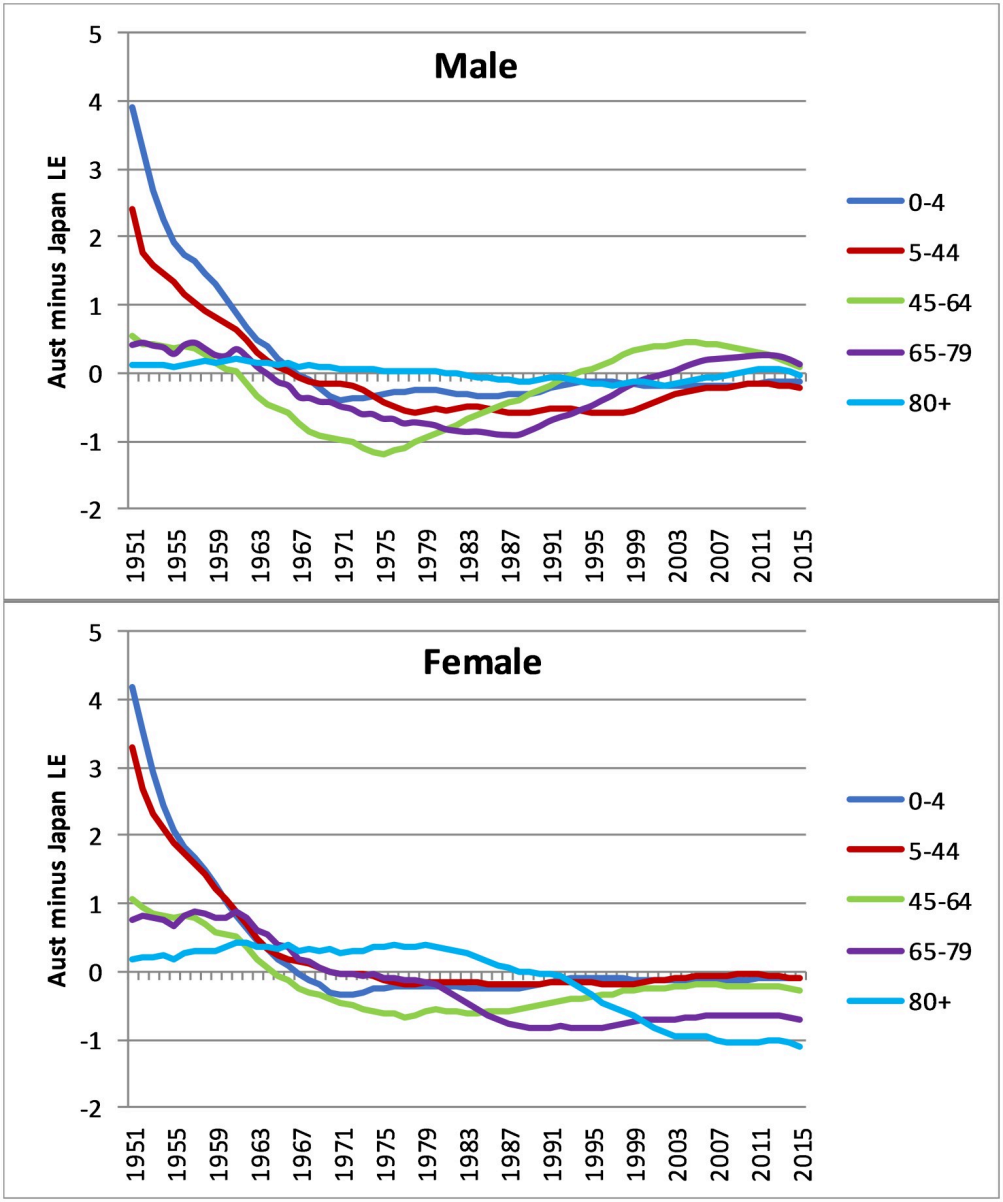
Age contribution to the difference in Australian and Japanese life expectancy, males and females, 1951–2015 (three-year average).

Most gains in Japanese compared with Australian life expectancy until 1960 were due to greater mortality reductions in Japan at ages less than 45 years, particularly less than five years (Fig 2). From 1960–75, age group 45–64 years made an increasingly greater net contribution to Japan’s male life expectancy, but this trend then reversed and from the mid-1990s it made a net contribution to Australia’s life expectancy. A similarly favourable trend for Australia is clear at ages 65–79 from the late 1980s. By 2016, all age groups’ contribution to the countries’ relative life expectancy was close to parity. For females, the Japanese mortality advantage at ages 45–64 has gradually reduced since the late 1970s, and by 2016 almost all of the higher life expectancy advantage for Japanese females was due to lower mortality at ages 65–79 compared with Australia and, especially, at 80 years and over where the contribution to life expectancy had widened to over one year.

### Contribution by birth cohort

**Fig 3:**
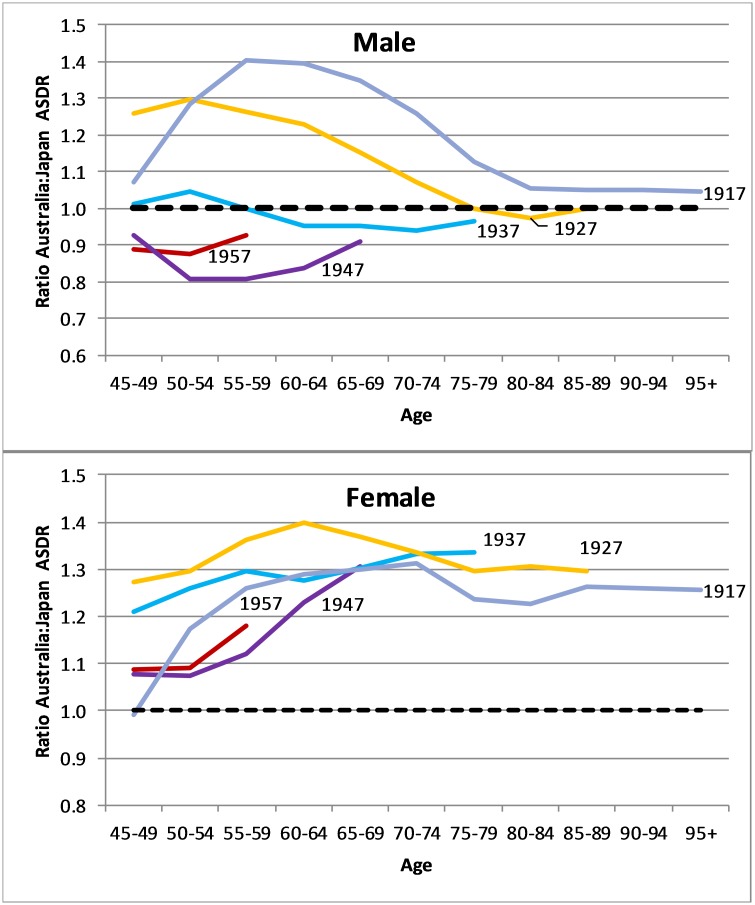
Ratio of Australia to Japan birth cohort age-specific death rates (ASDR), males and females, 1952–2016.

We can improve our understanding of favourable mortality trends in Australian males compared with Japan in recent decades by assessing changes in relative mortality at different ages according to birth cohort. Fig 3 shows that there has been a significant reduction in the ratio of Australian to Japanese male age-specific death rates (ASDRs) from males born in 1917 to those born in 1947, with the latter showing lower deaths rates in Australia compared to Japan at each age beyond 45 years. For the cohort born 10 years later (1957), however, there is evidence of a shift back to parity in death rates. For females, excess female mortality in Australia remained comparatively high across all ages in the earlier (1917–1937) birth cohorts (ASDR ratio of 1.2–1.4) (Fig 3). However there is evidence of lower excess death rates, around 10%, for Australian women at ages 45–54 in the more recent birth cohorts.

### Contribution of causes

**Fig 4:**
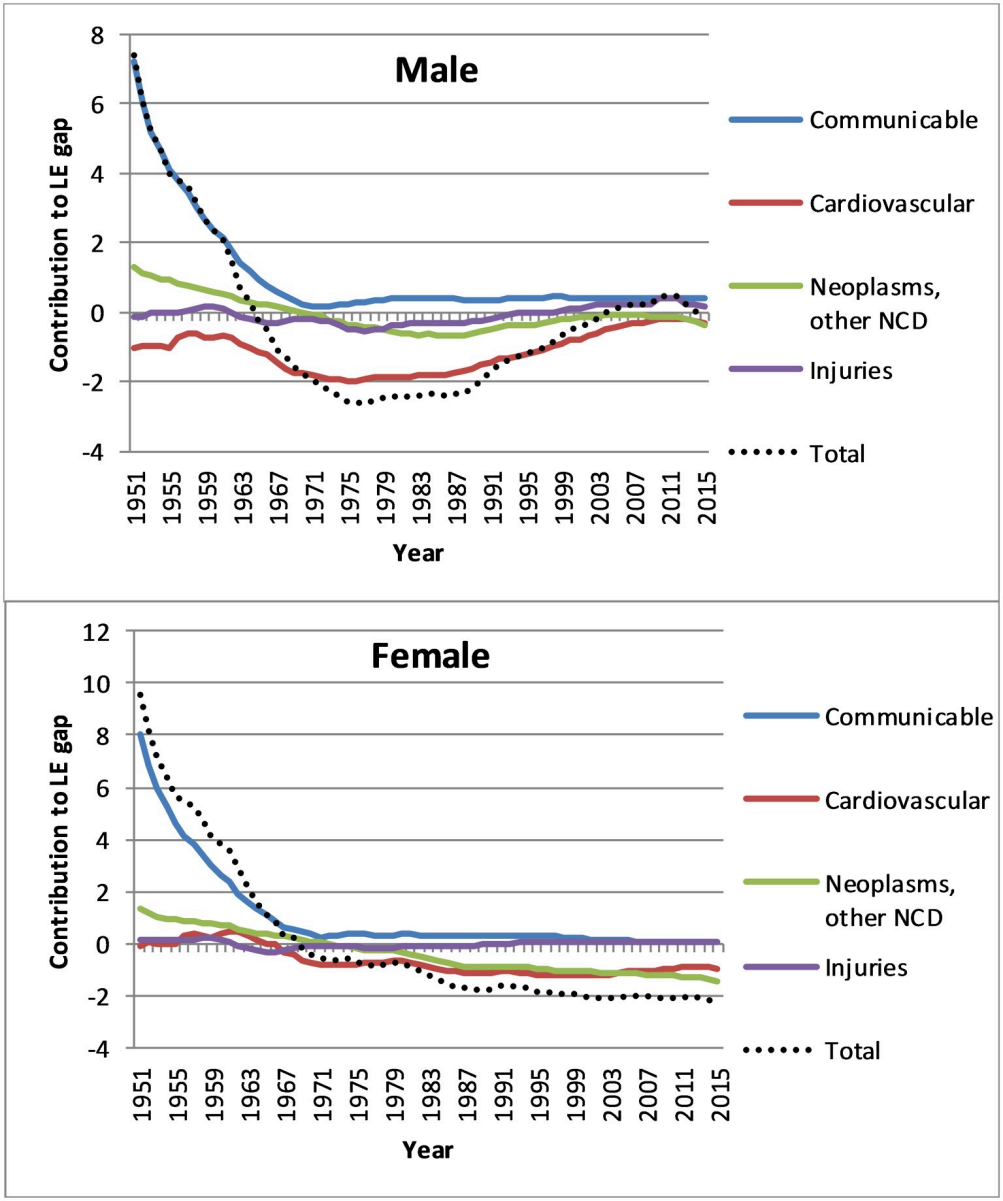
Contribution of major causes of death to the difference in Australian and Japanese life expectancy, males and females, 1951–2015 (three-year average).

The dramatic gains in Japanese compared with Australian life expectancy between 1950 and 1970 were due predominantly (just over three-quarters) to the much greater declines in communicable disease mortality in Japan. These conditions are typically most prevalent at younger ages and were already comparatively low in Australia at the start of the period (Fig 4, Table 1). The contribution of non-communicable diseases to Japanese life expectancy gains in this period was smaller but still significant, especially due to ischaemic heart disease (IHD).

**Table 1:** Contribution of specific causes of death to the difference in Australian and Japanese life expectancy, males and females, 1951–2015 (three-year average).

	**Contribution to LE difference (years)**					**Change in contribution to LE difference (years)**			
***Males*** **Cause**	**1951**	**1970**	**1990**	**2011**	**2015**	**1951–70**	**1970–90**	**1990–11**	**2011–15**
**Communicable**	**7.2**	**0.2**	**0.3**	**0.4**	**0.4**	**-7.0**	**0.1**	**0.0**	**0.0**
Lower respiratory infections	1.2	0.1	0.5	0.5	0.5	-1.1	0.4	0.0	0.0
Diarrheal diseases, TB	4.3	0.4	0.0	0.0	0.0	-3.9	-0.4	0.0	0.0
Other communicable	1.7	-0.3	-0.2	-0.1	-0.1	-2.0	0.1	0.0	0.0
**Non-communicable**	**0.3**	**-1.8**	**-2.1**	**-0.3**	**-0.7**	**-2.1**	**-0.3**	**1.8**	**-0.4**
Stomach cancer	0.7	0.8	0.6	0.4	0.4	0.0	-0.2	-0.1	0.0
Liver cancer	0.0	0.0	0.4	0.2	0.2	0.0	0.4	-0.1	0.0
Lung cancer	-0.1	-0.4	-0.2	0.1	0.1	-0.2	0.1	0.3	0.0
Other neoplasms	-0.4	-0.3	-0.7	-0.5	-0.6	0.1	-0.4	0.2	-0.1
Ischaemic heart disease	-2.1	-3.3	-1.8	-0.5	-0.6	-1.2	1.5	1.3	-0.1
Cerebrovascular disease	1.3	1.6	0.5	0.4	0.3	0.3	-1.2	-0.1	-0.1
Other cardiovascular	-0.3	-0.1	-0.2	0.0	-0.1	0.2	-0.1	0.1	0.0
Diabetes	0.0	0.0	-0.1	-0.2	-0.2	0.0	0.0	-0.1	0.0
Chronic respiratory	0.1	-0.3	-0.3	-0.2	-0.2	-0.4	0.0	0.2	0.0
Dementia	0.0	0.0	0.1	0.2	0.3	0.0	0.1	0.2	0.0
Other non-communicable	1.1	0.2	-0.4	-0.2	-0.3	-0.9	-0.5	-0.1	-0.1
**Injuries**	**-0.1**	**-0.2**	**-0.2**	**0.3**	**0.2**	**-0.1**	**0.0**	**0.5**	**-0.1**
Self-harm	0.3	0.0	0.0	0.3	0.2	-0.3	0.0	0.3	-0.1
Transport injuries	-0.7	-0.3	-0.2	-0.1	-0.1	0.4	0.1	0.1	0.0
Other injuries	0.3	0.1	0.0	0.1	0.1	-0.2	-0.1	0.1	0.0
**Total**	**7.4**	**-1.8**	**-2.0**	**0.3**	**-0.1**	**-9.2**	**-0.2**	**2.3**	**-0.5**
	**Contribution to LE gap (years)**					**Change in contribution to LE gap (years)**			
***Females*** **Cause**	**1951**	**1970**	**1990**	**2011**	**2015**	**1951–70**	**1970–90**	**1990–11**	**2011–15**
**Communicable**	**8.1**	**0.4**	**0.3**	**0.1**	**0.1**	**-7.7**	**-0.1**	**-0.2**	**0.0**
Lower respiratory infections	1.4	0.3	0.4	0.1	0.1	-1.1	0.1	-0.2	0.0
Diarrheal diseases, TB	4.6	0.3	0.0	0.0	0.0	-4.3	-0.2	0.0	0.0
Other communicable	2.1	-0.2	-0.1	0.0	0.0	-2.3	0.0	0.0	0.0
**Non-communicable**	**1.3**	**-0.6**	**-2.0**	**-2.2**	**-2.4**	**-1.9**	**-1.4**	**-0.2**	**-0.2**
Lung cancer	0.0	0.0	-0.1	-0.2	-0.2	0.0	-0.1	-0.1	0.0
Breast cancer	-0.2	-0.4	-0.4	-0.2	-0.2	-0.1	0.0	0.2	0.0
Stomach cancer	0.4	0.6	0.4	0.2	0.2	0.3	-0.3	-0.2	0.0
Other neoplasms	0.0	-0.2	-0.4	-0.3	-0.4	-0.2	-0.1	0.0	-0.1
Ischaemic heart disease	-1.1	-2.1	-1.4	-0.6	-0.6	-1.0	0.7	0.8	0.0
Cerebrovascular disease	1.3	1.4	0.3	-0.2	-0.2	0.1	-1.1	-0.5	0.0
Other cardiovascular	-0.3	0.0	0.0	-0.1	-0.1	0.2	0.0	-0.1	0.0
Chronic respiratory	0.2	0.0	-0.2	-0.4	-0.4	-0.2	-0.3	-0.1	0.0
Diabetes	-0.1	-0.1	-0.1	-0.2	-0.2	0.1	0.0	-0.1	0.0
Dementia	0.0	0.0	0.1	0.4	0.4	0.0	0.1	0.3	0.0
Other non-communicable	1.1	0.2	-0.2	-0.6	-0.7	-1.1	-0.3	-0.4	-0.1
**Injuries**	**0.2**	**-0.1**	**0.0**	**0.1**	**0.0**	**-0.3**	**0.1**	**0.1**	**0.0**
Self-harm	0.3	0.1	0.1	0.2	0.2	-0.2	0.0	0.0	0.0
Other injuries	-0.1	-0.2	-0.1	-0.1	-0.2	-0.1	0.1	0.1	0.0
**Total**	**9.5**	**-0.4**	**-1.7**	**-2.0**	**-2.3**	**-9.9**	**-1.3**	**-0.3**	**-0.2**

Note: Communicable category includes communicable, neonatal, maternal and nutritional diseases. LE: Life expectancy.

From 1970–90, a period of negligible change in the gap between Australian and Japanese male life expectancy, most striking changes were that differential mortality trends from IHD favoured Australia, while the reverse was true for stroke, due to the much larger reductions in (the much higher initial) mortality from this cause in Japan. Differential mortality trends (ie larger declines) from non-communicable diseases contributed to almost all of the gain in Japanese female life expectancy over this period, with trends for IHD and cerebrovascular disease exhibiting similar patterns as for men.

From 1990–2011, just over half of Australia’s 2.3 years gain in male life expectancy was due to relatively faster declines in IHD death rates, with more rapid declines in mortality from lung conditions (lung cancer and chronic respiratory disease) and self-harm contributing to most of the remaining change. This was offset by Australia having slower declines in cerebrovascular disease mortality. Since 2011, there has been half a year gain in life expectancy among males in Japan, due almost entirely to lower death rates from non-communicable diseases such as IHD (a reversal of the long-term decline in the Japanese advantage over Australia), cerebrovascular disease and cancers. Transport injuries have made a negligible contribution to the male life expectancy difference over the past 25 years.

The continued widening of the Japanese female life expectancy advantage since the early 1990s has also been largely due to more favourable mortality trends from non-communicable diseases, particularly cerebrovascular disease and chronic respiratory diseases, while trends in IHD and dementia have been more beneficial for women in Australia. Mortality from IHD, chronic respiratory and cancers (aside from stomach cancer) accounted for about three-quarters of the life expectancy advantage of Japanese over Australian women in 2016. A clear difference with males is that cerebrovascular disease contributes to life expectancy advantage in Australian males but disadvantage in Australian females.

## Discussion

The considerable gains in Australian male life expectancy from the early 1970s led to Australia having slightly higher male life expectancy in Japan from 2005 to 2014, a demographic phenomenon that is perhaps not widely appreciated. This trend was mainly driven by mortality declines at ages 45–79 years from cardiovascular diseases, especially IHD, as well as from lung cancer and chronic respiratory diseases, conditions related to a number of risk factors, including smoking [18]. During this period, compared with other high-income countries, Australia had relatively strong and Japan relatively weak declines in mortality [2]. Australia’s male IHD mortality, which in 1970 was the second-highest mortality rates in the industrialised world began to decline thereafter [19]. This has been variously attributed to improved diet, particularly from lower saturated fat intake, and its effect on improving control of blood pressure and cholesterol, as well as declining smoking prevalence [7]. Australia experienced a rapid decline in smoking prevalence over many decades, starting in the mid-1960s with the release of the first major Reports on the health effects of smoking, which in turn has led to very substantial falls in mortality from major vascular diseases, chronic respiratory diseases, lung and other cancers [8,9,20,21]. More favourable trends in Australian compared with Japanese life expectancy were also due to the comparatively high death rate from suicide in Japan since the 1990s (where the age-standardised suicide death rate exceeds that of all other high-income countries, apart from South Korea); economic recession and increasing unemployment have been identified as key contributors to this excess mortality [2,5,22]. A clear demographic difference between the two countries during this period is the that Australia experienced far more in-migration that Japan; previous research has shown that the life expectancy of overseas-born Australian males was 0.4 years higher than Australian-born males, so the impact on overall trends is likely to be minor.[23]

The gains in Australian male life expectancy were most evident for the cohort born in and around 1947 and, to a lesser extent, in 1937 and 1957. This is consistent with findings from Japan of high cohort effects on mortality of those born around World War II [24]. This cohort in Japan has had particularly high smoking prevalence, while their counterparts in Australia benefitted from earlier declines due to the impact of a range of public health interventions starting in the 1960s [9,25].

In recent years Australia’s small advantage in male life expectancy has been reversed, with Japan gaining half a year of life expectancy since 2011 compared with Australia. The large mortality reduction benefits to Australia from smoking declines may well be over, as evidenced by there being no further contribution to life expectancy differences from lung cancer. Smoking prevalence among males in Australia is currently half that of Japan (16% compared with 27% in 2015); although male smoking prevalence in Japan has also fallen in recent decades, there is obviously much greater scope than in Australia for further declines, which is likely to translate into greater reductions in mortality from smoking-related causes in the future [26]. Importantly, the long-term trend of relatively lower mortality in Australian compared with Japanese males for progressively younger cohorts was reversed since the 1957 cohort, suggesting that the comparative mortality advantage among Australians born post-war may well be over.

In particular, the long term narrowing in excess mortality from IHD among Australian compared to Japanese men (and women), which began in the 1970s, appears to have halted around 2011, with recent evidence suggesting that the male IHD mortality disadvantage in Australia compared with Japan, is widening. It is yet too early to be confident if this is the beginning of a longer term period of stable, or possibly widening of relative IHD death rates between Australia and Japan, but on the basis of recent trends, albeit only for four years or so, the half century of larger relative mortality declines from IHD in Australia compared with Japan may be coming to an end.

While the main reasons behind this reversal are undoubtedly multifactorial, obesity may be a significant factor; the prevalence of overweight and obesity has worsened significantly in Australia in recent decades and is much higher than Japan, and current evidence suggests that mortality risk from chronic diseases for the extreme obese are similar to that for smokers [27, 28]. However, the long-term population level impact of obesity trends on mortality is remains unclear, with the possibility that the more extreme hazards of obesity might be mitigated through more effective treatment of its consequences, including diabetes and major vascular diseases. What is more certain is that future changes in life expectancy will be increasingly affected by survival trends at ages 80 years and over as this population group becomes more and more significant. This, together with the greater potential among Japanese men to reduce smoking prevalence further than in Australia, might well lead to a return of the comparative Australian disadvantage in IHD death rates among men not seen for some decades.

Factors other than smoking are also likely to contribute to current life expectancy differences between Australia and Japan. Cerebrovascular disease makes a net positive contribution to Australian life expectancy but did not experience similar trends as IHD in recent decades, despite its similar risk factors such as smoking, high blood pressure, high cholesterol and high salt intake [2]. Lower respiratory infection mortality, which predominantly occurs among the elderly, has also shown a persistent advantage to Australia, possibly due to lower Australian smoking prevalence. Stomach cancer has shown a moderate advantage for Australia, most likely because of higher salt intake in the past in Japan [29]. The lower mortality from dementia in Australia compared with Japan is more difficult to explain, in part because prevalence data are largely used to estimate mortality due to the likelihood of extensive misdiagnosis of dementia in mortality data [2]. The substantial disparity in reported dementia mortality rates in old age in the two countries’ vital registration systems illustrate the effect that different certification practices can have on resulting statistics. While the methods employed by the GBD are the most advanced to address these issues, there remains some uncertainty about causes of death in old age, where quality of cause of death certification is particularly problematic.[30]

The female advantage in life expectancy in Japan versus Australia has been, and remains, substantial; the relatively strong recent performance of female compared with male life expectancy in Japan is not observed in most other high-income countries [31]. Notably, cerebrovascular disease mortality among Japanese women is lower than in Australia, in contrast to males. Smoking prevalence among Japanese women remains low, and at the same time, most of the benefits of smoking control in Australian women appear to have been realised [26]. The comparatively low prevalence of other key risk factors, such as obesity, with its attendant health effects, harmful alcohol use and diabetes, is not likely to lead to a narrowing of the life expectancy advantage that Japanese women currently enjoy over other countries, including Australia. There are good reasons to expect that Japan’s female life expectancy advantage over Australia and other countries will continue in the immediate future, at least until the comparatively healthier older Japanese women die out. However, while these epidemiological trends are not necessarily propitious for Australia, the observation that almost all of the life expectancy advantage for Japanese females arises from their lower mortality at the oldest ages is at least suggestive of a convergence in life expectancy at birth with Australia over the next two decades or so as this oldest age group dies out, and assuming no major risk factor reversals or widening health inequalities among women in Australia.

## Supporting information

S1 Text ICD codes for specific causes of death. (DOCX)

S1 Data Deaths, causes of death and population, Australia and Japan, 1950–2016. (XLSX)
