# Supplementary material for: The setting of the rising sun? A recent comparative history of life expectancy trends in Japan and Australia
Source: PLoS One. 2019 Mar 28;14(3):e0214578. doi: 10.1371/journal.pone.0214578 (PMC6438604; doi:10.1371/journal.pone.0214578)
Supplement: S1 Text — (DOCX) [file pone.0214578.s001.docx]

**S1 Text. ICD codes for specific causes of death [1]**

Lower respiratory infections: J09-J11, J13, J14, J12.1, J12 (except J12.1), J15-J22, J85, P23

Diarrheal diseases, TB: A00, A02-A04, A06-A09, A15-A19, B90, P37.0

Lung cancer: C33-C34

Breast cancer: C50

Stomach cancer: C16

Ischaemic heart disease: I20-I25

Cerebrovascular disease: I60-I63 I65-I67, I69.0, I69.1, I69.2, I69.3

Chronic respiratory diseases: J40-J44, J47, 60-J65, J45-J46, D86.0, D86.2, D86.9, J84, J30- J39, J66-J70 (except J69), J82, J92, J93.0, J93.1, J95, J98 (except J98.1, J98.2, J98.3, J98.9), B18, I85, K70, K71.7, K72.1-K72.9, K73-K74, K75.2-K75.9, K76.6-K76.7, K76.9

Dementia: F00-F03, G30-G31

Self-harm: X70, X76-X77, X72-X74, X68, X71, X75, X78-X83, X60- X67, X69

[1] GBD 2015 Mortality and Causes of Death Collaborators. Global, regional, and national life expectancy, all-cause mortality, and cause-specific mortality for 249 causes of death, 1980–2015: a systematic analysis for the Global Burden of Disease Study 2015. Lancet. 2016; 388: 1459–1544.
